# The course of depression in late life as measured by the Montgomery and Asberg Depression Rating Scale in an observational study of hospitalized patients

**DOI:** 10.1186/s12888-015-0577-8

**Published:** 2015-08-05

**Authors:** Tom Borza, Knut Engedal, Sverre Bergh, Jūratė Šaltytė Benth, Geir Selbæk

**Affiliations:** Centre for Old Age Psychiatric Research, Innlandet Hospital Trust, Sanderud, PO Box 68, 2312 Ottestad, Norway; Institute of Clinical Medicine, Faculty of Medicine, University of Oslo, Oslo, Norway; Norwegian National Advisory Unit on Ageing and Health, Vestfold Hospital Trust, Tønsberg, Norway; Institute of Clinical Medicine, Campus AHUS, University of Oslo, Oslo, Norway; HØKH, Research Centre, Akershus University Hospital, Lørenskog, Norway

## Abstract

**Background:**

Depression and depressive symptoms are highly prevalent in old persons but are potentially reversible. Full recovery is the main goal in the treatment of depressive episodes. Compared to clinical trials, observational studies of patients with depression in late life (DLL) show poorer prognoses in terms of response and remission. However, observational studies on the course of DLL are scarce.

The aims of this study were to examine the course of DLL in terms of response, remission and symptom-specific changes as measured by the Montgomery and Asberg Depression Rating Scale (MADRS), and to explore which clinical variables were associated with the response and remission.

**Methods:**

This is an observational, multicenter and prospective study of patients aged 60 years and older who were referred to treatment of depression in the department of old-age psychiatry at specialist health care services in Norway. The patients were evaluated with the MADRS at admission to and discharge from hospital. The mean, median, minimum and maximum values for days stayed in hospital were 68, 53, 16 and 301, respectively.

Effect size (ES) was calculated to determine which MADRS symptoms changed most during the treatment. To assess the predictors for change in the MADRS score (continuous variable) and for remission and response (both dichotomous variables), regression models adjusting for cluster effects within center were estimated.

**Results:**

Of 145 inpatients, 99 (68.3 %) had a response to treatment (50 % or more improvement of the MADRS score). Remission (MADRS score ≤9 at discharge) was experienced in 74 (51.0 %) of the patients. Of the individual MADRS items, “reported sadness” (ES =0.88) and “lassitude” (ES = 0.80) showed the greatest amount of improvement, and “concentration difficulties” (ES = 0.50) showed the least amount of improvement during treatment. Having a diagnosis of dementia was associated with a lower remission rate and less improvement in the MADRS score during the treatment. Poorer physical health was associated with a lower response rate. Having experienced previous episode(s) of depression was associated with a lower remission rate.

**Conclusions:**

Recurrent episodes of depression, poor somatic health and a diagnosis of dementia were found to be negative prognostic factors for the course of DLL. Clinicians should therefore pay close attention to these factors when evaluating treatment.

**Trial registration:**

ClinicalTrials.gov NCT01952366.

## Background

Depression and depressive symptoms are highly prevalent in old persons and pose global challenges [1]. Depression in late life (DLL) is associated with medical comorbidity, dementia, functional impairment, reduced quality of life, suicidal behavior, increased mortality and increased service utilization [2–6].

Depressive episodes in old persons are potentially reversible, and full recovery is the main goal of treatment [7]. Compared to clinical trials, prospective observational studies of inpatients have shown lower recovery rates from depressive episodes in old persons, varying from 38–69 % [8, 9]. Studies have also shown that 50 % of patients with DLL can be partial- or non-responders to initial treatment [7]. However, patients with DLL have the same response and remission rates with pharmacotherapy and electroconvulsive therapy (ECT) as younger depressed patients, but relapse rates are higher [10]. A clinical profile of an old depressed patient with conditions such as comorbid anxiety, psychotic symptoms, poor self-esteem, poor sleep, medical comorbidity and coexisting cognitive impairment is associated with a poorer prognosis in terms of response and remission [7, 11]. Partial response to treatment of depressive episodes increases the risk of poor outcome in terms of relapses, medical comorbidity and suicide [11]. Guidelines for diagnosing and managing DLL [12] as well as strategies for improving recovery rates have been developed [7].

DLL often shows a heterogenous symptom profile [11, 13]. A study of symptoms of DLL showed that the items “depressed mood” and “loss of interest in work and activities” showed the most improvement on the Hamilton Rating Scale for Depression (HRSD) during treatment [14].

The Montgomery and Asberg Depression Rating Scale (MADRS) is designed to be sensitive to treatment effects [15]. The scale has high inter-rater reliability and is a valid tool for use in populations with DLL [15–17]. Several studies have used the MADRS to evaluate the treatment effects of DLL [9, 17, 18].

In Norway, most of the patients with DLL are treated in primary care. However, patients who do not respond to treatment in primary care and those with high medical complexity, diminished ability to take care of themselves, or severe depression (e.g. depression with psychosis or a high risk of suicide) can be referred for treatment to departments of old-age psychiatry in specialist health care as inpatients. Such departments of old-age psychiatry are well-established throughout the country and typically offer services to catchment areas with 15,000 to 30,000 older people above the age of 65 years. The number of beds at these departments varies, but is recommended to be between 0.8 and 1.2 beds per 1,000 older people [19].

According to the latest edition of the Oxford textbook of Old Age Psychiatry, purely observational data on the course of DLL are scarce [20], and we are not aware of any such study from the Nordic countries.

It is essential to have knowledge about how the individual depressive symptoms of DLL change during treatment [14]. The assessment of depressive symptoms is dependent on the scale that is used. To our knowledge, no previous study has focused on the symptom-specific changes as measured by MADRS during the treatment of DLL. To learn more about the course of DLL in hospitalized patients we have conducted a multicenter, prospective observational study entitled “Prognosis of Depression in the Elderly” (PRODE) in Norway. The aims of this study were 1) to examine the course of DLL during the stay in hospital in terms of response and remission at discharge, 2) to identify which clinical predictors were associated with these outcomes, and 3) to investigate which individual depressive symptoms showed greatest amount of change during treatment, as measured by the MADRS.

## Methods

### Design

PRODE is an observational Norwegian multicenter prospective study. Nine departments of old-age psychiatry (Innlandet Hospital Trust; Sanderud and Reinsvoll, Vestre Viken Hospital Trust; Lier, St. Olav Hospital; University Hospital of Trondheim; Oslo University Hospital; Ullevaal and Aker, Haukeland University Hospital; Bergen, Diakonhjemmet Hospital; Oslo and Stavanger University Hospital; Stavanger) participated in the study. The collection of data was performed by health professionals working in these departments. Standardized assessment scales were used. Assessors received standardized training prior to the study period and again twice a year during the study period in order to secure reliable data.

### The patients

Patients were eligible for inclusion if they were 60 years or older and had been referred to treatment for depression in the departments of old-age psychiatry at specialist psychiatric health services. Patients with life-threatening diseases as well as patients with dementia who had severe aphasia were excluded from the study.

### Attrition

The entire PRODE sample included 169 patients. In the present study, 24 patients were excluded (nine because they were outpatients and 15 due to incomplete MADRS records), leaving 145 inpatients in this study. There were no differences between the excluded and included patients regarding demographic and clinical characteristics as described in Table 1, except for marital status (there was a greater amount of single people among the included patients) and duration of depression (more patients with a duration of current depression of 13 weeks or more among the excluded patients).

**Table 1:** Demographic and clinical characteristics of the sample (*n* = 145, if not specified)

Variable	Value
Age, mean (SD)	75.9 (6.7)
Female, n (%)	106 (73.1)
Years of education (*n* = 138), mean (SD)	10.1 (3.1)
Days of stay, mean (SD)	68.3 (46.8)
Marital status	
- Not-single (married/living together), n (%)	62 (42.8)
- Single (widow(er)/divorced/living apart), n (%)	83 (57.2)
GMHR^1^ categories (%)	
- Good (very good/good)	74 (51.0)
- Poor (fair/poor)	71 (49.0)
Cognitive function, mean MMSE^2^ (*n* = 140), mean (SD)	25.9 (3.6)
MMSE, categories (*n* = 140)	
- MMSE < 26 (%)	40.7
- MMSE ≥ 26 (%)	59.3
Diagnosis of dementia, n (%)	13 (9.0)
Number of drugs, mean (SD)	5.9 (3.1)
Number of psychotropic drugs, mean (SD)	2.1 (1.4)
Activities of daily living (IADL)^3^ (*n* = 143)	
- IADL per item score, mean (SD)	0.69 (0.26)
HADS-A^4^, mean (SD) (*n* = 141)	11.5 (4.7)
Age at onset of the first lifetime depressive episode, in categories (*n* = 140)	
- <60 years (%)	48.6
- ≥60 years (%)	51.4
Duration of depressive episode, categories (*n* = 142)	
- <13 weeks (%)	47.2
- ≥13 weeks (%)	52.8
Previous depressive episode(s), n (%)	101 (69.7)
Mean MADRS^5^ score (SD) at inclusion	26.1 (8.6)
Bipolar diagnosis (ICD-10), n (%)	10 (6.9)
Depression with psychosis (ICD-10), n (%)	15 (10.3)
Patients with personality disorder (ICD-10), n (%)	3 (2.1)

^1^GMHR = General Medical Health Rating Scale

^2^MMSE = Mini Mental Status Examination

^3^IADL = Lawton Instrumental Activities of Daily Living Scale

^4^HADS = Hospital Anxiety and Depression Scale

^5^MADRS = Montgomery and Asberg Depression Rating Scale

### Representativeness

To assess representativeness, five of the nine study centers were able to collect data on age and gender from all eligible patients invited to participate in the study, and also to those who declined to participate. In these five study centers, 159 patients were approached and 34 refused to participate in the study. There were no differences in age or gender between those who participated in the study and those who refused to participate.

### Procedures

Data was collected from December 2009 to July 2013. Patients were assessed for eligibility and included as early as possible after admission. The mean number of days from admission to inclusion was 5.7 days (SD = 6.0). Patients were diagnosed with depression and dementia according to the criteria of the Tenth Revision of the International Classification of Diseases and Health Related Problems (ICD-10) [21]. Discharge from the department of old age psychiatry was not settled according to a standard procedure, but according to the clinical procedure of the individual departments. The discharge assessment was carried out as near as possible to the discharge date.

### Measurements

Information on prior depressive and psychiatric history, including the number of previous depressive episodes and age at onset of the first lifetime depressive episode, was obtained from case notes and structured interviews with the patients and caregivers. The MADRS, a measurement of the severity of depression, was the primary outcome in the study. The MADRS consists of 10 items, each rated from 0 points (no symptoms) to 6 points (severe symptoms), with higher score denoting more severe depression [15].

Cognitive function was evaluated by the Mini-Mental-State Examination (MMSE), (score range 0–30) [22]. A higher score indicates better cognition.

Anxiety symptoms were assessed by the seven-item anxiety portion of the Hospital Anxiety and Depression Scale (HADS-A) (score range 0–21) [23]. A higher score denotes more severe symptoms.

Physical health was rated with the General Medical Health Rating scale (GMHR), a four-point (excellent, good, fair, and poor) global rating scale for medical overall comorbidity. The GMHR takes into account each patient’s number of general medical conditions, the severity of those conditions and the patient’s use of medications [24]. For the analyses, the GMHR was dichotomized to good (excellent/good) and poor (fair/poor).

The instrumental activities of daily living (IADL) were evaluated by using the Lawton and Brody scale [25]. This scale consists of five items for males and eight for females. Each item was dichotomized (0 or 1) in line with the original publication [25]. We generated a mean score by dividing the IADL sum score by 5 for males and 8 for females. A higher score denotes a higher level of functioning (maximum score is 1 and minimum is 0).

Marital status was dichotomized to not-single (including married or living together) and single (including widow [er], divorced or living apart).

Age at the onset of the first lifetime depressive episode was dichotomized to 59 or earlier and 60 or later, as described in other studies [26]. The number of previous depressive episodes was dichotomized to no previous depressive episodes or previous depressive episode(s). The duration of the current depressive episode was dichotomized to 12 weeks or shorter and 13 weeks or longer.

We classified the medications used according to the Anatomical and Therapeutic Chemical (ATC) classification system. In the ATC system, the active substances are divided into different groups according to the organ or system on which the substances act and their therapeutic, pharmacological and chemical properties. Dosages of the various psychotropic drugs were calculated by using Defined Daily Dose (DDD), the World Health Organization’s (WHO) statistical measure of drug consumption that gives the assumed average maintenance dose per day for a drug that is used for its main indication in adults. The use of the ATC classification system and DDD as a measuring unit is recommended by the WHO for drug utilization studies [27].

Data on the MADRS and the patient’s use of medications were collected at the time of patients’ inclusion in the study and at discharge from the department. Data on the GMHR, IADL, HADS-A and MMSE were collected at inclusion.

### Outcomes

In line with previous recommendations for outcomes in clinical studies of depression [28, 29] and an observational study of DLL using the MADRS, [9], remission was defined by a score of ≤ 9 on the MADRS at the time of discharge. Response was defined as a reduction of at least 50 % in the MADRS score from time of inclusion to discharge [18, 28].

### Treatment

Patients were not assigned to a particular treatment protocol. Treatment depended on patients’ needs and practices at the local study center. Treatment was multidisciplinary, and virtually all patients received a mix of individualized medication therapy, environmental therapy, different psychotherapeutic approaches, physical exercise and social-/ economic oriented facilitation.

The mean, median, minimum and maximum values for days of stay in hospital were 68.3, 53.0, 16, and 301, respectively. The use of psychotropic drugs at time of inclusion and discharge is shown in Table 2. Thirty-eight of the 145 patients (26.2 %) were treated with ECT. The mean number of ECT treatments was 12.7 (SD = 6.2). Other types of treatment were not rigorously quantified. However, 31 % had individualized psychotherapy, 100 % had individual supportive-based conversations, 92 % had focus on social/- economic facilitation, 97 % had regular physical exercise as part of the treatment and 63 % participated in some kind of group therapy.

**Table 2:** Use of psychotropic drugs at inclusion and discharge (*n* = 145)

Psychotropic drug:	Inclusion (%):	Discharge (%:)	Mean DDD^1^ (SD^2^), inclusion:	Mean DDD^1^ (SD^2^), discharge:
Antidepressants	74.5	84.1	1.48 (0.91)	1.74 (0.93)
Anxiolytics	22.1	20.0	0.51 (0.35)	0.38 (0.29)
Hypnotics	38.6	36.6	1.01 (0.51)	0.92 (0.26)
Antipsychotics	23.4	27.6	0.42 (0.46)	0.38 (0.26)
Antidementia drugs	2.1	7.6	0.89 (0.38)	0.80 (0.37)
Lithium	4.8	6.9	0.57 (0.24)	0.52 (0.21)
Antiepileptics	9.0	13.1	0.77 (0.54)	0.57 (0.49)
Antiparkinson drugs	2.8	2.1	0.52 (0.22)	0.67 (0.33)

^1^DDD = Defined daily dose

^2^SD = Standard deviation

### Statistical analysis

Data was analysed using the Statistical Program for Social Science package (SPSS v. 22.0) and Statistical Analysis System (SAS v. 9.3).

Independent samples *t*-test or *χ*^2^-test were used to assess the differences in age and gender between the patients who refused to participate and those patients who were included in the PRODE study, and to compare the clinical and demographic characteristics between the excluded and included patients in the analyses of this paper. The change in use of psychotropic drugs from inclusion to discharge was assessed using McNemar’s test. The *t*-test for paired samples was applied to assess the change in DDD and MADRS score from inclusion to discharge.

Some records of the IADL items were missing in 22 of the 145 patients. Missing scores on IADL items were imputed for patients with 50 % or fewer missing values. The imputation was performed separately for males and females by drawing one random number per missing value from the distribution of a specific item, estimated on the available data.

Effect size (ES) was calculated to determine which MADRS symptoms changed most during the treatment. The use of ES for this purpose has previously been described [30]. In the present analyses we used Cohen’s d. As the study was performed concurrently at several different centers, a hierarchical structure might be present in the data. According to an intra-class correlation coefficient, the porportion of the variance within a centre (cluster effect) was estimated to vary between 0.6 % and 12.0 % for MADRS symptoms. Hence, the standard deviation (SD) was adjusted for cluster effect by estimating an empty mixed linear model with random effects for intercepts (SAS MIXED procedure). The ES (Cohen’s d) for the specific MADRS symptoms was calculated by dividing the mean change in score by the SD, which was adjusted for cluster effect. Effect sizes greater than 0.80 are considered large, while those between 0.50 and 0.79 are moderate, and those less than 0.50 are small [31].

To assess the predictors for change in the MADRS score (continuous variable) and for remission and response (both dichotomous variables), regression models adjusting for cluster effect caused by sampling from several centers were estimated. A linear mixed model with random effects for intercepts was estimated for continuous outcomes (SAS MIXED procedure). For dichotmous outcomes, logistic regression models for hierarchical data with random effects for intercepts were fitted (SAS GLIMMIX procedure). Bivariate models were first estimated for each predictor (MMSE, HADS-A, number of drugs, IADL score, previous depressive episode(s), diagnosis of dementia, age at onset of the first lifetime depressive episode, duration of current depressive episode, GMHR and psychotic depression) and confounder (age, years of education, gender, marital status and days of stay in hospital). Then multivariable models, including all predictors and confounders, were estimated. Akaike’s Information Criterion (AIC) was then applied to eliminate the redundant predictors. A smaller value of AIC means a better model. Results with p-values below 0.05 were considered statistically significant.

### Ethical and legal considerations

The participating patients and caregivers were given oral and written information, and they subsequently gave consent to participate in writing. For patients without the capacity to give consent, their next of kin had to give consent in writing on behalf of the patient. The study was approved by the Regional Committee of Medical Research Ethics and the Privacy and Data Protection Officer at Oslo University Hospital. Trial registration: ClinicalTrials.gov NCT01952366.

## Results

Of the 145 patients included in the study, 106 (73.1 %) were females. The mean age for all participants was 75.9 (SD = 6.7) years. The mean age was 76.2 (SD = 7.0) for females and 75.1 (SD = 5.8) for males. Further demographic and clinical characteristics of the study population are shown in Table 1. None of the patients died or withdrew their consent during the study period.

At the time of inclusion in the study 74.5 % of the patients used antidepressants, compared to 84.1 % at discharge (*p* = 0.02). In addition, 2.1 % used anti-dementia drugs at inclusion, compared to 7.6 % at discharge (*p* = 0.02). No significant changes in the use of other psychotropic drugs from inclusion to discharge were found. Psychotropic drugs were used by 91.7 % and 95.2 % of patients at inclusion and at discharge, respectively (*p* = 0.27). The DDD among those patients using antidepressants increased from 1.48 (SD = 0.91) at inclusion to 1.74 (SD = 0.93) at discharge (*p* = 0.001). For other psychotropic drugs, there were no differences in DDD among those using the drug at both time points.

At inclusion, two of the 145 patients (1.4 %) resided in a nursing home. Of the remaining 143 patients living in their own homes at inclusion, 50 (35.0 %) received domiciliary care. At inclusion, 57 of 140 patients (40.7 %) had a MMSE score of 25 or lower. Thirteen of the 145 patients (9.0 %) had a diagnosis of dementia established during their stay. The mean MMSE score at inclusion among these patients was 20.1 (SD = 3.6). Fifteen of the 145 patients (10.3 %) were discharged to a nursing home, 125 (86.2 %) to their homes and five (3.4 %) to “other”, e.g. patients receiving care as inpatients at a lower level of care. Seventy-five of the patients (51.7 %) received further psychiatric treatment from specialist health care as outpatients after discharge.

The mean MADRS score decreased from 26.1 (SD = 8.6) at inclusion to 10.7 (SD = 7.9) at discharge (*p* < 0.001). According to our definitions of response and remission, 74 of the 145 patients (51.0 %) experienced a remission, and 99 (68.3 %) responded to treatment. Of the 99 patients who responded to treatment, 31 (31.3 %) were not in remission at discharge. Of the total study population of 145 patients, 68 (46.7 %) both responded to treatment and experienced a remission.

The mean MADRS item scores at inclusion, the change in mean MADRS item scores, and the effect sizes for the MADRS items are all presented in Table 3. The MADRS items of “reported sadness” (ES = 0.88) and “lassitude” (ES = 0.80) showed the greatest effect sizes and “concentration difficulties” (ES = 0.50) showed the lowest effect size during treatment.

**Table 3:** The mean score, change in the mean score, intra-class correlation coefficient for change in the mean score, standard deviation for change in the mean score and effect size of the individual Montgomery and Asberg Depression Rating Scale (MADRS) items during treatment (*n* = 145)

MADRS-item:	Mean score at inclusion	Change (decrease) in mean score	ICC^1^ for change in mean score	SD^2^ for change in score adjusted for cluster effect	ES^3^
Apparent sadness	2.55	1.49	4.7	2.08	0.72
Reported sadness	2.98	2.00	12.0	2.26	0.88
Inner tension	3.05	1.53	7.4	2.33	0.66
Reduced sleep	2.41	1.58	1.3	2.65	0.60
Reduced appetite	2.09	1.46	0.6	2.23	0.65
Concentration difficulties	2.73	1.29	6.5	2.56	0.50
Lassitude	3.19	1.86	7.9	2.33	0.80
Inability to feel	2.59	1.57	9.2	2.33	0.67
Pessimistic thoughts	2.89	1.57	0.6	2.46	0.64
Suicidal thoughts	1.69	1.12	9.3	1.45	0.77

^1^ICC = Intra-class correlation coefficient

^2^SD = Standard deviation

^3^ES = Effect size

Results of the regression models for change in the MADRS score, response and remission as dependent variables are presented in Table 4. The predictors in the regression models correlated weakly, except for (higher) age at onset of the first lifetime depressive episode with (no) previous depressive episode(s) (Pearson’s correlation coefficient = 0.65) and having a diagnosis of dementia with a lower MMSE score (Pearson’s correlation coefficient = 0.54). According to the bivariate linear mixed model, having a diagnosis of dementia and lower score on MMSE were associated with less improvement of the MADRS score. Only having a diagnosis of dementia was associated with lower levels of improvement in the multivariable analysis. In the logistic models, poorer physical health was associated with a lower response rate in both the bivariate and the multivariable analyses. An age of < 60 years at onset of the first lifetime depressive episode, having previous depressive episode(s), a higher HADS-A score and lower IADL functioning were associated with a lower remission rate in the bivariate analysis. Having a diagnosis of dementia and previous depressive episode(s) were associated with a lower remission rate in the multivariable analysis. Among the confounding variables, a longer stay in hospital (higher number of days) was associated with a lower response rate in the multivariable analysis and a lower remission rate in the bivariate and multivariable analyses.

**Table 4:** Regression models with associated predictors for change in Montgomery and Asberg Depression Rating Scale (MADRS) score, response and remission as dependent variable (*n* = 122)

	Linear mixed model (Change in MADRS score as dependent variable)				Logistic regression model for hierarchical data (Response as dependent variable)				Logistic regression model for hierarchical data (Remission as dependent variable)			
	Bivariate analysis		Multivariable analysis		Bivariate analysis		Multivariable analysis		Bivariate analysis		Multivariable analysis	
	Coefficient (95 % CI^2^)	p-value	Coefficient (95 % CI^2^)	p-value	OR^3^ (95 % CI^2^)	p-value	OR^3^ (95 % CI^2^)	p-value	OR^3^ (95 % CI^2^)	p-value	OR^3^ (95 % CI^2^)	p-value
MMSE	0.49 (0.03;0.96)	0.038*			1.10 (0.98;1.23)	0.094			1.05 (0.95;1.17)	0.337		
HADS-A	0.14 (−0.25;0.52)	0.482			0.97 (0.88;1.06)	0.443			0.90 (0.83;0.99)	0.024*	0.93 (0.84;1.03)	0.150
Number of drugs	0.10 (−0.47;0.68)	0.720			0.90 (0.79;1.03)	0.117			0.95 (0.84;1.08)	0.445		
Previous depressive episode (s) (Yes as 0^1^)	−0.09 (−3.80;3.63)	0.964	−2.01 (−7.09;3.07)	0.436	1.28 (1.28;3.14)	0.581			3.30 (1.41;7.68)	0.006*	4.61 (1.64;13.01)	0.004*
IADL score	2.11 (−4.81;9.04)	0.547	0.60 (−7.99;6.79)	0.873	2.67 (0.56;12.79)	0.217			5.36 (1.06;27.27)	0.043*	3.82 (0.63;23.20)	0.144
Diagnosis of dementia (No diagnosis as 0^1^)	−8.26 (−14.19;-2.33)	0.007*	−9.03 (−15.40;2.64)	0.006*	0.27 (0.07;1.05)	0.058	0.25 (0.06;1.03)	0.055	0.21 (0.04;1.08)	0.061	0.11 (0.02;0.73)	0.023*
Age at onset of first lifetime depressive episode (<60 years as 0^1^)	1.10 (−2.29;4.48)	0.523	3.35 (− 1.28;7.99)	0.154	1.79 (0.80;4.00)	0.157			2.50 (1.17;5.36)	0.019*		
Duration of current depressive episode (≤12 weeks as 0^1^)	−2.61 (−6.22;1.00)	0.154	−2.40 (−6.14;1.35)	0.207	0.83 (0.35;1.96)	0.667			0.56 (0.24;1.33)	0.184	0.50 (0.20;1.28)	0.147
GMHR (Very good/good as 0^1^)	−2.23 (−5.67;1.21)	0.202	−1.38 (−4.90;2.13)	0.436	0.35 (0.15;0.79)	0.012*	0.38 (0.16;0.90)	0.029*	0.52 (0.24;1.11)	0.091		
Depression with psychosis according ICD-10 (no as 0^1^)	3.42 (−2.68;9.52)	0.269	3.42 (−2.86;9.69)	0.283	1.05 (0.24;4.62)	0.946			1.68 (0.42;6.70)	0.458		
Age	−0.15 (−0.41;0.11)	0.258	−0.28 (−0.57;0.006)	0.055	1.06 (0.99;1.14)	0.064	1.04 (0.97;1.12)	0.232	1.01 (0.95;1.07)	0.731	1.00 (0.93;1.07)	0.983
Years of education	0.06 (−0.53;0.64)	0.850	-0.19 (-0.78;0.41)	0.533	0.91 (0.80;1.04)	0.160	0.92 (0.80;1.05)	0.218	0.95 (0.84;1.08)	0.438	0.94 (0.82;1.09)	0.413
Gender (Female as 0^1^)	2.58 (−1.26;6.42)	0.187	1.82 (−2.09;5.73)	0.357	0.71 (0.29;1.74)	0.449	0.81 (0.31;2.15)	0.675	1.16 (0.49;2.75)	0.733	0.96 (0.36;2.58)	0.931
Marital status (Not-single as 0^1^)	−2.28 (−5.69;1.14)	0.189	−2.25 (−5.84;1.33)	0.216	1.20 (0.54;2.69)	0.656	1.01 (0.42;2.40)	0.989	1.24 (0.58;2.66)	0.580	0.86 (0.34;2.17)	0.752
Days of stay in hospital	−0.02 (−0.06;0.01)	0.216	−0.03 (−0.07;0.004)	0.083	0.99 (0.98;1.00)	0.061	0.99 (0.98;1.00)	0.042*	0.989 (0.979;0.999)	0.026*	0.987 (0.976;0.997)	0.014*

^1^ = Reference category

^2^CI = Confidence interval

^3^OR = Odds ratio

A sensitivity analysis excluding those patients with a diagnosis of dementia was performed. The significant findings in our regression models were not altered.

## Discussion

In the present study on the course of depression in hospitalized patients, 74 of the 145 patients (51.0 %) experienced a remission, defined as a total MADRS score of nine or lower at discharge. A treatment response (a reduction of 50 % or more of the MADRS score) was found in 99 (68.3 %) patients. These figures are comparable to the results from similar studies [8, 9], however the results from prospective observational studies of the prognosis of hospitalized patients with DLL vary largely [9]. Our study differs from other observational studies of DLL in that patients with dementia are included, as we wanted the study population to be as representative as possible of the clinical practice of old-age psychiatry in specialist health care services in Norway.

The main goal in treatment of depressive episodes of DLL is to attain remission. The remission rate was relatively low at discharge in the present study, and we suggest that there could be several explanations as to why we and other observational studies of patients with DLL have found these low remission rates.

Firstly, low remission rates could be related to the nature of DLL. Our study population included many patients with DLL who were difficult to treat in primary care. About half of the patients had comorbidities that resulted in poor physical health. Nine percent had dementia, and 40.7 % had a MMSE score below 26 at inclusion. Our population of old people could present unspecific symptoms (such as reduced sleep and reduced appetite) and symptoms of cognitive impairment (like concentration difficulties) that the MADRS tapped as depressive, rather than as symptoms of comorbidities, thus making it harder to attain remission as measured by the MADRS.

Secondly, the low remission rate could be related to the treatment offered. The treatment was perhaps not intensive or long enough for all patients. Several patients were referred to further treatment in specialist psychiatric health care after discharge, but at a lower level of care than they had received during the study period. Some of these patients might have reached remission after discharge from departments of old-age psychiatry. The fact that treatment was continued at a lower level of care when the patients were substantially better, but not in complete remission, may indicate a well-functioning health service.

Thirdly, the explanation for a relatively poor outcome in terms of remission rates could be related to the use of the MADRS and the definitions of remission and response. Our definition of remission is in line with previous recommendations [29], but it could be a conservative measure for inpatients treated for DLL at departments for old-age psychiatry in specialist health care [18]. A Norwegian study of the MADRS as a screening tool for DLL found that the best cut-off was 13/14, as compared to the ICD-10 criteria for depression [16].

Ultimately, the period of time around discharge can be a vulnerable period for patients with DLL, and may yield symptoms that the MADRS taps.

### Symptom-specific changes

The highest mean scores at the time of inclusion were observed for the MADRS items “lassitude”, “inner tension” and “reported sadness”, while the lowest was for the item “suicidal thoughts.” The effect size of the symptom-specific effects of treatment is a descriptive, not inferential measure of change [30]. The MADRS items “reported sadness” (ES = 0.88) and “lassitude” (ES = 0.80) showed the greatest improvement while “concentration difficulties” showed the smallest improvement (ES = 0.50) during treatment in the present study. In a multicenter, placebo-controlled study of 728 patients with DLL, Nelson et al. applied symptom-specific effect sizes and examined which symptoms on the Hamilton Depression Rating Scale (HRSD) changed the most during Sertraline treatment. “Depressed mood,” and “decreased interest and activity” improved the most, while “loss of weight” improved the least [14]. The study excluded patients with psychotic or bipolar disorders, a diagnosis of dementia, or a MMSE score less than 24. Thus, even though the study population and assessment scale differ from the present study, the results are comparable. The symptoms equivalent to the ICD-10 core symptoms of a diagnosis of depression (depressed mood to a degree that is definitely abnormal for the individual, loss of interest or pleasure in activities that are normally pleasurable, and decreased energy or increased fatigability [21]) improved the most during treatment of DLL [11, 14]. Previous studies indicate that this may apply to other age groups as well [14, 30]. The present study showed that “concentration difficulties” improved the least. This may be explained by the fact that a considerable proportion of the current sample also had cognitive impairment or dementia, in which concentration difficulty is a common symptom. From our findings, it seems that concentration difficulty can be a symptom of DLL that improves to a lesser extent during treatment.

### Clinical predictors

The three regression models yielded different results regarding the association between clinical predictors and the outcomes; the multivariable analyses showed that having a diagnosis of dementia was associated with less improvement in MADRS score; poorer physical health was associated with a lower response rate; and having a diagnosis of dementia and previous depressive episode(s) were associated with a lower remission rate. This underlines that response (assessed as continuous or dichotomous variables) and remission are different outcomes. Indeed, we found that 31.3 % of the responders did not experience a remission.

It can be difficult to distinguish between the effects attributable to the number of depressive episodes and those attributable to age at the first lifetime onset of depressive episode [26]. In our data these two predictors exhibited a strong association (Pearson’s correlation coefficient of 0.65). Having previous depressive episode(s) became the strongest predictor of a lower remission rate in the multivariable analysis. Driscoll et al. have described that when recurrent episodes of depression occurred, they most likely were more complicated to treat than the first episode of depression among DLL patients [32]. A recent study has described that a first lifetime onset of depression in early life (EOD) was associated with more severe depressive symptoms and more residual symptoms over time, and that a first lifetime onset of depression in later life (LOD) was associated with more cognitive and neurological changes [17]. However, the definitions of EOD and LOD, study populations, and the assessment of outcomes all differ, and there is no clear consensus in the literature of how a patient’s age at the onset of first lifetime depression predicts outcome in depressive episodes [17, 26, 33–36]. Our findings confirm previous studies showing that recurrent episodes of depression among DLL patients might be more difficult to treat.

DLL and comorbid anxiety commonly coexist. Studies have found that a greater severity of anxiety symptoms can have a negative impact on acute treatment response in late-life depression [11, 37, 38]. Our findings on the association between anxiety and a lower remission rate in the bivariate analysis might support the view that attaining remission in DLL patients with severe anxiety can be difficult. However, this association between anxiety and remission was not significant in the multivariable analysis.

In the present study, worse physical health was significantly associated with a worse outcome in terms of response. Our finding is in line with previous findings, showing that a high medical burden can complicate the treatment of DLL [2, 8, 11].

In addition, having a diagnosis of dementia was strongly associated with less improvement in the MADRS score during treatment. Having a diagnosis of dementia was also associated with lower remission in the multivariable analysis. As only 9.0 % of the study population had a diagnosis of dementia, it is hard to draw any clear conclusions, but the results may indicate that patients with dementia were less responsive to treatment. Previous studies have described decreased treatment response in patients with depression and dementia [39]. Some possible reasons for this relationship can be that depression in patients with dementia may have a different neurobiology from depression in those without dementia. Further, it is difficult to define homogenous groups of patients with depression and dementia. Also, there is evidence that subgroups of patients with cognitive impairment (e.g. with a severe burden of white matter hyperintensities) have unfavorable responses to antidepressants [11, 40]. Cognitive impairments in DLL could persist even after remission of depression [41] and symptoms of cognitive impairments and DLL can overlap.

### Limitations and strengths

The study population was heterogeneous, as there was a great variation in the degree of depressive symptoms as measured by the MADRS, cognitive impairment, and the days of the patients’ stay in hospital. Thus, we treated the days of patients’ stay in hospital as a confounder in the analysis. The study was observational, and treatment was not standardized but varied according to local treatment regimen and patients’ different needs. It is therefore difficult to generalize the results to a specific group of DLL patients.

Some of the study centers could not include patients for the entire duration of the study period, due to reorganization of health services.

The study was performed in a multicenter setting with different assessors, which could influence inter-rater reliability. However, the MADRS is shown to have good inter-rater reliability among different health professionals [15], and this scale is recommended for use in assessing depression in Norway [42]. Furthermore, the assessors participated in structured training in the use of assessment scales both prior to and during the study. Moreover, the statistical models used have been adjusted for the possible cluster effect due to the multicenter design.

The MADRS has the best validity for patients without dementia or with mild dementia [16]. In the present study population there were 13 patients with a diagnosis of dementia; and among these the mean MMSE score was 20.1 (SD = 3.6). Thus, the MADRS would be a valid tool to assess DLL in most patients in this study.

The mean number of days from admission to inclusion was 5.7 (SD = 6.0). It could be that for some of the patients, the depressive symptoms had changed between the time of admission and the time they were assessed.

A strength of the study is the use of well-established assessment scales. In order to reflect clinical, everyday practice, the inclusion criteria were broad, and the design of the study was observational and prospective. Thus, the study will have high clinical relevance for patients with DLL who are admitted to hospital.

## Conclusions

This observational study of DLL in hospitalized patients showed that at discharge the response rate was higher than the remission rate, according to our definitions. “Reported sadness” and “lassitude” were the items on the MADRS that showed the greatest amount of improvement during treatment.

Recurrent episodes of depression, poor somatic health, and a diagnosis of dementia were found to be important negative prognostic factors for the course of DLL. Clinicians should pay close attention to these factors when planning and evaluating treatment.

## Abbreviations

MADRS: Montgomery and Asberg Depression Rating Scale
DLL: Depression in late life
ECT: Electroconvulsive therapy
HRSD: Hamilton rating scale for depression
PRODE: Prognosis of depression in the elderly
ICD-10: Tenth Revision of the International Classification of Diseases and Health Related Problems
MMSE: Mini-mental-state examination
HADS: Hospital anxiety and depression scale
GMHR: General medical health rating scale
IADL: Instrumental activities of daily living
ATC system: Anatomical and therapeutic chemical classification system
DDD: Defined daily dose
ES: Effect size
AIC: Akaike’s information criterion
SD: Standard deviation
EOD: Early onset of first lifetime depression
LOD: Late onset of first lifetime depression
